# Partial liver resection alters the bile salt-FGF19 axis in patients with perihilar cholangiocarcinoma: Implications for liver regeneration

**DOI:** 10.1097/HC9.0000000000000445

**Published:** 2024-06-05

**Authors:** Kiran V.K. Koelfat, Frank G. Schaap, Kim M.C. van Mierlo, Martin Leníček, Ilka Sauer, Gregory van der Kroft, Anjali A.J. Röth, Jan Bednarsch, Iakovos Amygdalos, Georg Lurje, Maxime J.L. Dewulf, Sven A. Lang, Ulf P. Neumann, Steven W.M. Olde Damink

**Affiliations:** 1 Department of General, Visceral and Transplant Surgery, University Hospital RWTH Aachen, Aachen, Germany; 2 Department of Surgery, Maastricht University Medical Centre & NUTRIM School of Nutrition and Translational Research in Metabolism, Maastricht University, Maastricht, The Netherlands; 3 Institute of Medical Biochemistry and Laboratory Diagnostics, First Faculty of Medicine, General University Hospital in Prague, Charles University, Prague, Czech Republic

## Abstract

**Background:**

Extended liver resection is the only treatment option for perihilar cholangiocarcinoma (pCCA). Bile salts and the gut hormone FGF19, both promoters of liver regeneration (LR), have not been investigated in patients undergoing resection for pCCA. We aimed to evaluate the bile salt-FGF19 axis perioperatively in pCCA and study its effects on LR.

**Methods:**

Plasma bile salts, FGF19, and C4 (bile salt synthesis marker) were assessed in patients with pCCA and controls (colorectal liver metastases), before and after resection on postoperative days (PODs) 1, 3, and 7. Hepatic bile salts were determined in intraoperative liver biopsies.

**Results:**

Partial liver resection in pCCA elicited a sharp decline in bile salt and FGF19 plasma levels on POD 1 and remained low thereafter, unlike in controls, where bile salts rose gradually. Preoperatively, suppressed C4 in pCCA normalized postoperatively to levels similar to those in the controls. The remnant liver volume and postoperative bilirubin levels were negatively associated with postoperative C4 levels. Furthermore, patients who developed postoperative liver failure had nearly undetectable C4 levels on POD 7. Hepatic bile salts strongly predicted hyperbilirubinemia on POD 7 in both groups. Finally, postoperative bile salt levels on day 7 were an independent predictor of LR.

**Conclusions:**

Partial liver resection alters the bile salt-FGF19 axis, but its derailment is unrelated to LR in pCCA. Postoperative monitoring of circulating bile salts and their production may be useful for monitoring LR.

## Graphical abstract

**Figure:**
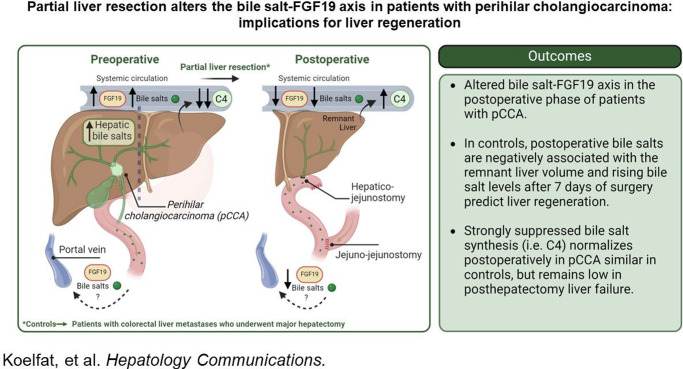

## INTRODUCTION

Liver regeneration (LR) is a vital and complex process for recovery from hepatic injury or partial resection of the liver tissue.^1^ A perturbed enterohepatic circulation inflicted by obstructive cholestasis is believed to impair postoperative liver regrowth.^2^,^3^ For patients with perihilar cholangiocarcinoma (pCCA), a rare biliary malignancy obstructing the extrahepatic biliary tree and generally causing obstructive cholestasis, extensive resection is often the only curative option.^4^ Unfortunately, postoperative mortality in pCCA patients remains high (up to 19%) and is mostly caused by post-hepatectomy liver failure (PHLF).^5^,^6^,^7^,^8^ Thus far, enterohepatic bile salt signaling, which promotes LR in rodents, has not been addressed in the context of LR in humans in (post)cholestatic patients.

Bile salts are crucial in gut-liver signaling and promote LR by activating the key bile salt-sensing receptor farnesoid X receptor (FXR), which is primarily expressed in ileocytes and hepatocytes.^9^ Ileal FXR promotes LR by stimulating the production of the gut hormone FGF19 (Fgf15 in rodents).^10^ In preclinical models, Fgf15/FGF19 promotes LR indirectly by maintaining bile salt homeostasis and directly through mitogenic actions.^10^,^11^ FGF19 contributes to bile salt homeostasis by repressing the gateway biosynthetic enzyme cholesterol-7-α-hydroxylase (*CYP7A1*), with CYP7A1 activity reflected by plasma levels of 7-α-hydroxy-4-cholesten-3-one (C4, a stable intermediate). Together with the hepatic FXR-mediated control of bile salt extraction, synthesis, and biliary excretion, these homeostatic mechanisms strive to maintain intracellular bile salts at nontoxic levels.^10^,^12^,^13^,^14^ Hepatic bile salt overload is thought to contribute to the development of PHLF. In (chronic) cholestatic liver disease (eg, primary biliary cirrhosis, alcoholic hepatitis, and biliary atresia), the bile salt-FGF19 axis is altered and associated with repressed bile salt synthesis, probably to prevent further hepatic bile salt overload.^15^,^16^,^17^,^18^ The impact of partial liver resection on the bile salt-FGF19 axis in the regenerating (post)cholestatic liver has thus far remained unknown because of the paucity of human studies on this topic.^19^,^20^,^21^

Since enterohepatic circulation is abrogated in pCCA, we postulated that partial liver resection impacts gut-liver crosstalk mediated by bile salt/FGF19 signaling and consequently LR. We aimed to study the bile salt-FGF19 axis before and after partial liver resection in patients with pCCA, and its relationship with LR. Noncholestatic patients who underwent partial hepatectomy for colorectal liver metastases (CRLM) were included in the control group.

## METHODS

### Patients

This was a retrospective study with a pretest and posttest design using prospectively collected data from consecutive patients who underwent major liver resection (≥ 3 segments) for pCCA or CRLM at the Department of General, Visceral, and Transplantation Surgery of the University Hospital RWTH Aachen (Germany). All patients undergoing major liver resection were evaluated and included if relevant clinical/surgical data and preoperative and postoperative blood samples were available. Patients were excluded if a baseline (preoperative) blood sample was not available. Forty-six patients had paired CT volumetrics available and were included in the LR analyses. This study was conducted in accordance with the requirements of the Institutional Review Board of the RWTH Aachen University (Aachen, Germany), the Declaration of Helsinki, and the Good Clinical Practice Guidelines (ICH-GCP).

### Data collection and definitions

Clinical data were obtained from medical records and included patient characteristics, surgical outcomes, and preoperative and postoperative laboratory parameters (bilirubin, alkaline phosphatase, alanine aminotransferase, aspartate aminotransferase, gamma-glutamyl transferase, albumin, C-reactive protein, international normalized ratio), and postoperative complications.^22^ Postoperative major morbidity was classified as Clavien-Dindo grade IIIA or higher. Hyperbilirubinemia was defined as serum total bilirubin levels ≥1.2 mg/dL (20.5 µmol/L), according to local clinical guidelines. PHLF was scored according to the classification of the International Study Group of Liver Surgery.^23^ Relevant entries into the medical records of all patients were checked for up to 90 days after liver surgery. Pathology reports of the resected specimens were assessed for features of histological cholestasis.

### Blood samples, liver specimens, and laboratory analyses

Blood samples were collected after overnight fasting preoperatively and on postoperative days (PODs) 1, 3, and 7. EDTA plasma was stored at −80°C. Preoperative blood samples were collected from the radial artery line before the abdominal incision. Postoperative blood was collected from the radial artery line (POD 1) or peripheral venous catheter (PODs 3 and 7). Liver wedge biopsies of the excised liver specimen were taken at a large distance from the tumor by the pathologist, immediately frozen in liquid nitrogen, and stored at −80°C. Of note, liver biopsies were derived from nondrained liver segments in patients with pCCA who received preoperative unilateral biliary drainage (78% of cases) of the future remnant liver. FGF19 was determined by sandwich ELISA as described.^15^ Total bile salts in plasma or liver homogenates (normalized to liver wet weight) were measured according to the manufacturer’s protocol using an enzymatic cycling method (Diazyme Laboratories).^24^ Plasma levels of 7-α-hydroxy-4-cholesten-3-one (C4) were determined by liquid chromatography-mass spectrometry.^25^

### Liver volumetry and LR

The preoperative CT scan closest to the date of surgery and the first postoperative oncological control CT scan were used for the volumetric measurements. CT volumetric data were collected as part of the standard clinical management. Details of the technical assessments are reported elsewhere.^26^ The following variables were obtained (all in mL): functional total liver volume (calculated by subtracting the tumor volume, from the total liver volume), anticipated future remnant liver volume (RLV), and postoperative functional remnant liver volume (FRLV). LR was calculated using the following formula: (%)=[(FRLV at follow-up–RLV)/RLV]×100. See Supplemental Table S1, http://links.lww.com/HC9/A891 for details on the CT liver volumetry of all patients in both groups.

### Statistical analysis

Data are expressed as mean ± SD or median (interquartile range) with the 10th to 90th percentile. Longitudinal data are presented as mean ± SEM. Differences between groups were evaluated using the Mann-Whitney *U* test or Student *t* test, when appropriate. Categorical variables were analyzed using the Fisher exact test. Correlations were evaluated using the Spearman (ρ) correlation coefficient. One of the plasma samples from the control group had an unexplainable outlier of the FGF19 value (2.61 ng/mL); therefore, this value on POD 1 was excluded from further data analyses. The predictive accuracy of hepatic bile salts in discriminating between low and high bilirubin levels was evaluated using receiver operating characteristic curve analysis by calculating the AUC. To avoid subject deletion due to incomplete postoperative blood sampling, linear mixed model (LMM) analyses were conducted to study the postoperative plasma courses and postoperative (PODs 1–7) associations between RLV, bile salts, FGF19, C4, and bilirubin. Data derived from LMM analyses are reported as “estimate, 95% CI and *p* value,” and are interpreted as follows: one unit change of the predictor variable is associated with an increase of the positive estimate or decrease of the negative estimate, of the dependent variable. Univariate and multivariable regression analyses were performed to investigate their association with LR. First, we identified variables that were associated (*p* < 0.05) with LR, the dependent variable. These were then entered in the multivariable linear regression, with LR as the dependent variable. Model selection was based on the best model fit. Statistical analyses were performed using SPSS 29.0 (IBM SPSS Inc.) or Prism 9 (GraphPad Software Inc.).

## RESULTS

### Patients characteristics

A total of 23 patients with pCCA (mean age, 66 y; 43% female) were included and compared to a control group of 43 patients with CRLM (mean age, 58 y; 32% female). Baseline biochemical liver test results were considerably higher in patients with pCCA (bilirubin, aspartate aminotransferase, gamma-glutamyl transferase, and alkaline phosphatase) (Table 1). In the pCCA group, 18 patients (78%) underwent preoperative biliary drainage (viz. unilateral) of the future remnant liver, with 16 of these patients receiving a biliary stent. The clinical characteristics are listed in Table 1. All patients underwent major liver resection (≥3 segments). An overview of the surgical procedure is provided in Supplemental Table S2, http://links.lww.com/HC9/A891.

**TABLE 1:** Patient characteristics

Characteristic	CRLM (n=43)	pCCA (n=23)	*p*
Sex			
Male	29	13	0.584
Female	14	10	
Mean age (y)	58±11	66±9	**0.002**
Median BMI (kg/m^2^)	25.2 [22.5–28.5]	25.1 [22.1–28.5]	0.879
ASA 3 or 4			
No	15	13	0.76
Yes	28	10	
Preoperative laboratory values			
Bilirubin (µmol/L)	7 [5–11]	21 [11–60]	**<0.001**
AST (IU/L)	36 [28–45]	47 [33–90]	**0.010**
γGT (IU/L)	71 [35–183] (n=41)	423 [208–706] (n=22)	**<0.001**
AP (IU/L)	105 [80–196] (n=41)	286 [181–442] (n=22)	**<0.001**
Albumin (g/L)	4.1 [3.6–4.4] (n=26)	3.8 [3.3–4.1] (n=20)	0.061
CRP (mg/L)	6 [2–20] (n=41)	15 [8–53]	**0.003**
INR	0.99 [0.92–1.04]	1.06 [0.92–1.19] (n=22)	**0.003**
Preoperative biliary drainage			
No	43	5	**<0.001**
Yes	0	18	
PVE			
No	33	15	0.324
Yes	10	7	
Postoperative clinical outcome			
Post-hepatectomy liver failure (PHLF)			
No	42	17	**0.006**
Yes	1	6	
90-d mortality			
No	40	21	0.583
Yes	3	1	
Hospital admission (d)	11 [8–20]	20 [15–-27]	**0.002**
Major surgical morbidity (C-D III-V)			
No	24	13	0.582
Yes	19	10	

*Note*: Values depicted as mean [SD] or median [IQR]. Student *t* test, Chi square, or Mann-Whitney *U* test were used to test differences between groups.

Significant P-values (<0.05) are in bold.

Abbreviations: AP, alkaline phosphatase; ASA, American Society of Anesthesiologists; AST, aspartate aminotransferase; BMI, body mass index; C-D, Clavien-Dindo; CRLM, colorectal liver metastases; CRP, C-reactive protein; GGT, gamma-glutamyl transferase; INR, international normalized ratio; pCCA, perihilar cholangiocarcinoma; PHLF, post-hepatectomy liver failure; PVE, portal vein embolization.

### Preoperative alteration of the bile salt-FGF19-axis and suppressed bile salt synthesis in pCCA

**FIGURE 1:**
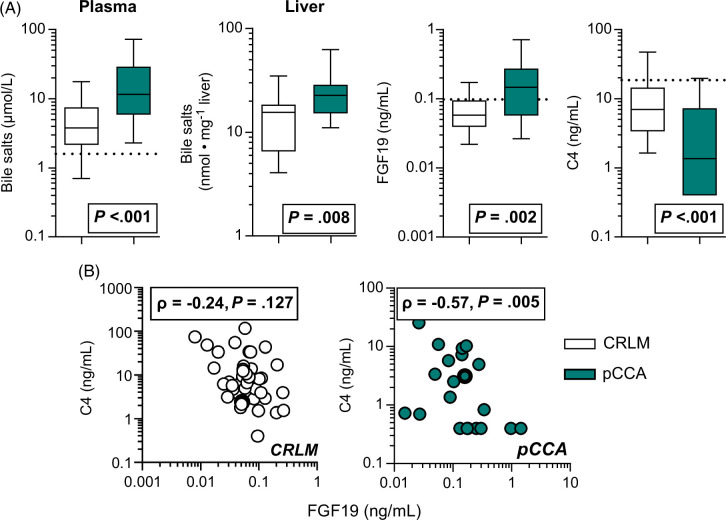
Modified gut-liver crosstalk propelled by altered bile salt-FGF19 axis in patients with pCCA. (A) Baseline values of systemic and liver bile salts, FGF19, and C4 in patients with pCCA (n=23) in comparison with CRLM controls (n=43). For reference, the dotted lines indicate the median values of bile salts (1.6 µmol/L), FGF19 (0.098 ng/mL), or C4 (18.7 ng/mL) in healthy subjects.^27^ (B) Correlations between FGF19 and C4 levels at baseline. Data are depicted as a box with 25th, median, and 75th percentile and the ends of the whiskers are the 10th to 90th percentile. Differences were evaluated by the Mann-Whitney *U* test. Correlations were evaluated with the Spearman (ρ) correlation coefficient. *p* values are provided in the graphs. Abbreviations: C4, 7α-hydroxy-4-cholesten-3-one; CRLM, colorectal liver metastases; pCCA, perihilar cholangiocarcinoma.

We first studied whether the regulatory bile salt-FGF19-axis was adapted at baseline in patients with pCCA. Bile salts and FGF19 in systemic plasma were all higher in the pCCA group than in the control group and were accompanied by elevated liver bile salts directly after resection (Figure 1A). This was paralleled by lower baseline C4 levels, which were inversely correlated with FGF19 in patients with pCAA, but not in controls (Figures 1A, B). Patients with histological features of cholestasis (n=9) had significantly elevated liver and plasma bile salt and FGF19 levels, in contrast to low C4 levels (Supplemental Figure S1A, http://links.lww.com/HC9/A891). Furthermore, markers of cholestatic and liver cell injury (bilirubin, alkaline phosphatase, and gamma-glutamyl transferase) were all significantly elevated in these patients (Supplemental Figure S1B, http://links.lww.com/HC9/A891).

### Postoperative divergent bile salt-FGF19 plasma time course in pCCA

**FIGURE 2:**
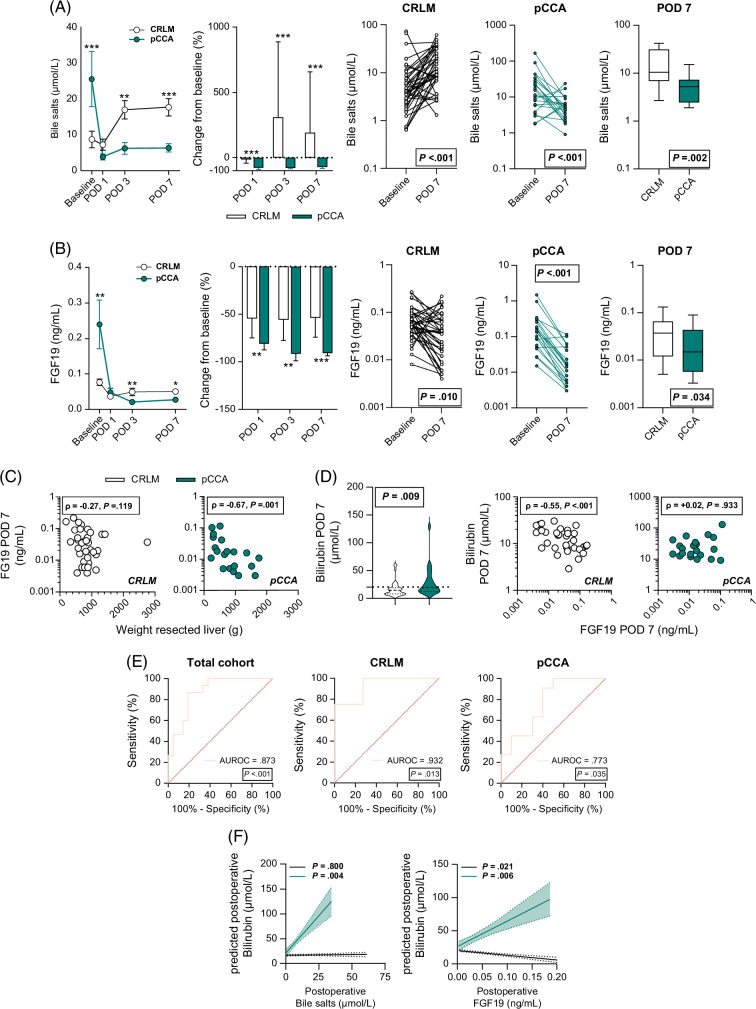
Divergent postoperative courses of bile salt and FGF19 in patients with pCCA. The dynamics of bile salts-FGF19 in pCCA versus CRLM were investigated at baseline (prior surgery) and PODs 1, 3, and 7. Postoperative time course and relative changes from baseline over time for (A) bile salts and (B) FGF19. (C) Correlation between the weight of the resected liver specimen and FGF19 level on POD 7. (D) Violin plots with the median and IQR of bilirubin levels on POD 7 in CRLM versus patients with pCCA and the correlation with FGF19 on POD 7. The dashed line denotes the threshold for normal bilirubin levels. (E) Receiver operating characteristic (ROC) curves evaluating the predictive performance of hepatic bile salts for hyperbilirubinemia on POD 7 in the total cohort (n=36), CRLM (n=15), and pCCA (n=21). (F) Linear mixed model analyses evaluating the effect of postoperative bile salts or FGF19 (fixed effects with random intercept) on the postoperative course of bilirubin levels. Black lines with standard error denote patients with CRLM and green lines with standard error denote patients with pCCA. The white symbols, bars, and boxes denote patients with CRLM and green symbols denote patients with pCCA. Differences between baseline and POD 7 were evaluated using the Wilcoxon matched-pairs signed rank sum test. Differences between the CRLM and pCCA groups were evaluated using the Mann-Whitney *U* test. *p* values are depicted. Asterisks indicate significance levels between patients with CRLM and pCCA: *p < 0.05, **p < 0.01, and ***p < 0.001. Abbreviations: CRLM, colorectal liver metastases; pCCA, perihilar cholangiocarcinoma; POD, postoperative day.

We previously showed that in patients with CRLM, systemic bile salts rise postoperatively and are positively associated with LR, in line with observations in preclinical studies.^19^,^28^,^29^ In the preoperative phase, the bile salt-FGF19 axis was altered in pCCA in response to the cholestatic environment, in contrast to controls. Because partial liver resection leads to a smaller RLV, and bile salts and FGF19 are key initiators of LR in preclinical models,^9^,^10^ we further studied the postoperative plasma time courses of the bile salt-FGF19-axis and its relationship to bilirubin (see Supplemental Table S3, http://links.lww.com/HC9/A891 for a detailed overview of the LMM analyses with estimates [95% CI] of the bile salt-FGF19 plasma time courses). After liver surgery, bile salt and FGF19 levels dropped sharply in patients with pCCA on the first POD and remained significantly lower on PODs 3 and 7 than at baseline (Figures 2A, B). In contrast, bile salt levels rose gradually over time, reaching a significant elevation on PODs 3 and 7 compared to baseline in the CRLM group (Figure 2A). Similar to patients with pCCA, although to a lesser extent, FGF19 levels were also significantly decreased on POD 1 in CRLM controls compared to baseline (0.058 [0.039–0.096] vs. 0.027 [0.014–0.052] ng/mL, *p* < 0.001) and remained stable thereafter (Figure 2B). However, FGF19 levels were significantly higher in CRLM controls than in the pCCA group on PODs 3 and 7 (Figure 2B). Notably, FGF19 levels on POD 7 showed a significant inverse correlation with the weight of the resected liver specimen in pCCA (Figure 2C).

Bilirubin levels remained higher in patients with pCCA at POD 7 than in controls and were negatively correlated with FGF19 levels in patients with CRLM, but not in patients with pCCA (Figure 2D). The postoperative plasma bilirubin time course is shown in Supplemental Figure S2, http://links.lww.com/HC9/A891. Bile salt overload causes hepatic injury and, in line with this, hepatic bile salts predicted hyperbilirubinemia on POD 7 in both groups (Figure 2E). Moreover, postoperatively elevated plasma bile salts were associated with increased postoperative bilirubin levels in pCCA, but not in CRLM controls (3.0, 95% CI [1.0, 5.0], *p* = 0.004 vs. 0.02, 95% CI [−0.2, 0.2], *p* = 0.800) (Figure 2F). Similarly, elevated postoperative FGF19 levels were significantly associated with rising bilirubin levels in pCCA (382, 95% CI [62, 701], *p* = 0.020), but with lower bilirubin levels in CRLM controls (−71, 95% CI [−121, −21], *p* = 0.006) (Figure 2F).

### Presuppressed bile salt synthesis rebounds after partial liver resection in pCCA

**FIGURE 3:**
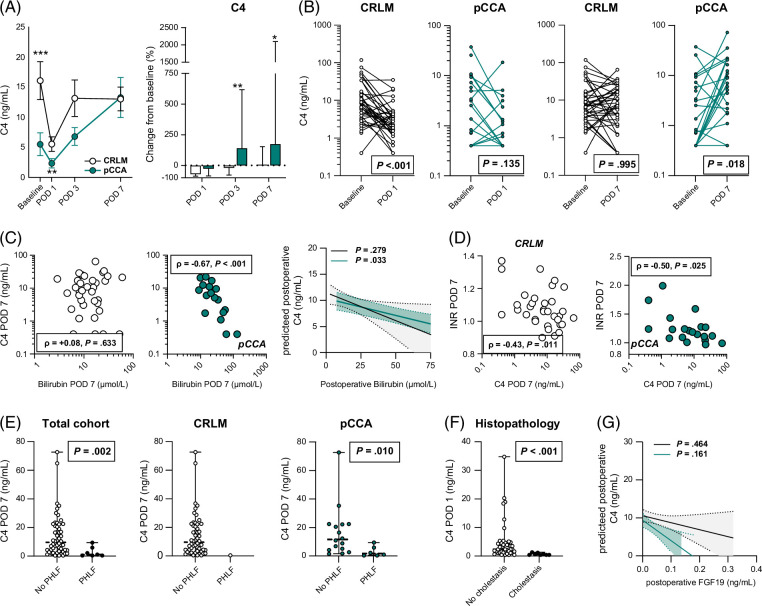
Postoperative recovery of bile salt synthesis in patients with pCCA. (A) The postoperative plasma course of the C4 reflects bile salt synthesis and the percentage change from baseline. (B) Paired analyses of C4 on POD 7 and C4 levels on POD 7 between patients with CRLM and pCCA. (C) Correlation between C4 and bilirubin levels on POD 7. (D) Relation between C4 and INR on POD 7. (E) Plasma C4 levels on day 7 of patients who did not develop PHLF compared with those who developed PHLF in the total cohort and subgroups (CRLM and pCCA). (F) C4 levels on POD 1 in histopathologically proven cholestasis (n=8, 1 patient had a missing C4 value on POD 1) versus noncholestatic liver parenchyma (n=41). (G) Linear mixed model analysis of the association between postoperative FGF19 and postoperative C4. Differences between baseline and POD 7 were evaluated by Wilcoxon matched-pairs signed-ranks sum test. Differences between the CRLM and pCCA groups were evaluated by the Mann-Whitney *U* test. Correlations were tested by Spearman rank correlation. *p* values are depicted. Asterisks indicate significance levels between patients with CRLM and pCCA: *p < 0.05, **p < 0.01, and ***p < 0.001. Abbreviations: C4, 7α-hydroxy-4-cholesten-3-one; CRLM, colorectal liver metastases; INR, international normalized ratio; pCCA, perihilar cholangiocarcinoma; PHLF, post-hepatectomy liver failure; POD, postoperative day.

Tight control of bile salt synthesis is critical to prevent hepatic bile salt overload–related toxicity, especially in the context of impaired partial liver secretion.^30^ Bile salt synthesis was already suppressed before surgery in patients with pCCA, but did not further decrease on POD 1 (*p* = 0.125), unlike in CRLM controls (*p* < 0.001) (Figure 3A). On POD 7, C4 levels were restored to preoperative values in both groups and did not differ between patients with CRLM and pCCA, although the absolute change from baseline to POD 7 was only significant in patients with pCCA (Figure 3B). Furthermore, bilirubin levels were negatively correlated with C4 levels on POD 7, but this was not observed in controls (Figure 3C). In fact, postoperative bilirubin levels predicted lower C4 levels in the postoperative phase of patients with pCCA than in controls (−0.07, 95% CI [−0.12, −0.01], *p* = 0.033 vs. −0.10, 95% CI [−0.29, 0.09], *p* = 0.279) (Figure 3C). Bearing in mind that normalized C4 could reflect a broad recovery of hepatic metabolic function, C4 levels on POD 7 were negatively related to international normalized ratio in both groups (Figure 3D), supporting this notion. In line with this, patients who developed PHLF in either group had significantly lower C4 levels on POD 7 than patients who did not develop PHLF (Figure 3E). Moreover, patients with histopathological features of cholestasis had near-undetectable C4 levels on POD 1 compared with patients without these features (Figure 3F). Finally, the postoperative change in FGF19 levels during the first week was not associated with postoperative C4 levels in either patient group (−18.2, 95% CI [−67.1, 30.8], *p* = 0.464 vs*. −*54.1, 95% CI [−130.2, 22.1], *p* = 0.161) (Figure 3G).

### The extent of partial liver resection impacts postoperative plasma bile salts and C4

**FIGURE 4:**
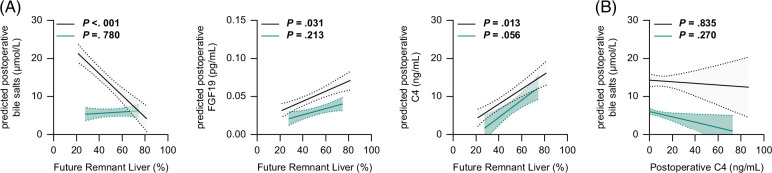
The relation between the future remnant liver and the postoperative plasma course of the bile salts, FGF19 and C4. Linear mixed model analyses were used to study the association between the remnant liver (%) and postoperative plasma course of (A) bile salts, FGF19, and C4. Lines are depicted with standard error; black lines denote patients with CRLM, and green lines denote patients with pCCA. (B) Association between postoperative C4 levels and bile salts during the postoperative phase. Abbreviations: C4, 7α-hydroxy-4-cholesten-3-one; CRLM, colorectal liver metastases; pCCA, perihilar cholangiocarcinoma.

A smaller remnant liver is associated with major postoperative morbidity in pCCA; therefore, strategies to optimize the future remnant liver are pivotal.^26^ Thus, we investigated whether the anticipated RLV affected postoperative plasma time courses of bile salts, FGF19, and C4, using LMM analyses. The calculated RLV was negatively associated with postoperative bile salts in controls, but not in pCCA (−0.285, 95% CI [−0.449, −0.121], *p* < 0.001 vs. 0.018, 95% CI [−0.109, 0.104], *p* = 0.780) (Figure 4A). In contrast, the calculated FRL had a positive correlation with postoperative C4 levels in controls and a positive trend in patients with pCCA (0.22, 95% CI [0.09, 0.35], *p* = 0.001 vs. 0.23, 95% CI [−0.01, 0.455], *p* = 0.056) (Figure 4A). The remnant liver also had a significant correlation with postoperative FGF19 levels in controls, but not in patients with pCCA (0.653, 95% CI [0.062, 1.243], *p* = 0.031 vs. 0.410, 95% CI [−0.244, 1.065], *p* = 0.213) (Figure 4A). Finally, the postoperative change in C4 was not associated with postoperative bile salt levels in either patient group (−0.02, 95% CI [−0.22, 0.18], *p* = 0.835 vs. −0.07 (−0.20, 0.06), *p* =0.270) (Figure 4B).

### Postoperative aberrant bile salt-FGF19 plasma response is not associated with LR in pCCA

As we found divergent plasma bile salt and FGF19 courses in the postoperative phase of pCCA and control patients, their association with LR was further investigated. For a total of 46 patients (CRLM controls, n=29; pCCA, n=17), sequential CT-liver volumetrics were available for LR analysis. Notably, apart from higher male sex in the group of patients without available CT-liver volumetrics, all other baseline characteristics were not different between patients with and without CT-liver volumetrics (Supplemental Table S4, http://links.lww.com/HC9/A891). The median follow-up time following liver surgery was 97 [IQR 39–216] days in patients with CRLM and 50 [IQR 29–150] days in patients with pCCA (*p* = 0.154). The starting point (anticipated %RLV) and actual liver volume recovery (%LR) were comparable between patients with CRLM and pCCA (Supplemental Table S1, http://links.lww.com/HC9/A891).

In the total cohort, bile salt levels on POD 7 were an independent predictor of LR, but FGF19 levels at any of the time points were not (Table 2). Further LMM analyses revealed that the postoperative mean change in bile salts was significantly associated with LR in controls but not in pCCA (1.2, 95% CI [0.2–2.1], *p* = 0.016 vs. 0.4, 95% CI [−1.4 to 2.2], *p* = 0.655). Age at the time of surgery, presence of pCCA, preoperative bilirubin, and C-reactive protein levels were also significant independent predictors of LR in univariable analysis (Table 2). Finally, in the multivariable analysis, bile salt levels on POD 7, corrected for age at the time of surgery, and preoperative bilirubin levels remained a significant positive predictor of LR (Table 2).

**TABLE 2:** Univariable and multivariable analyses of preoperative and postoperative variables associated with liver regeneration (%) in the total cohort

	Univariable analysis			Multivariable analysis		
Variable	Coefficient	95% CI	*p*	Coefficient	95% CI	*p*
Patient characteristics^†^						
Tumor type (pCCA reference)	−38.3	−74.0, −2.6	**0.036**			
Age (y)	−1.8	−3.4, −.25	**0.024**	−1.5	−3.0, −0.11	**0.036**
Sex (Female reference)	31.1	−4.0, 66.0	0.081			
BMI (kg/m^2^)	−0.8	−4.2, 2.6	0.634			
Weight resected liver specimen (g)	0.02	−0.03, 0.06	0.449			
Hospital admission (d)	−1.1	−2.4, 0.2	0.088			
Follow-up time (d)	0.01	−0.05, 0.07	0.675			
Bile salt pathway						
Liver bile salts (nmol/mg liver)	−1.2	−2.6, 0.3	0.112			
Bile salts (µmol/L)						
POD 1	2.7	−0.2, 4.6	**0.**073			
POD 3	0.8	−0.4, 2.0	0.184			
POD 7	2.1	0.9, 3.3	**0.001**	1.7	0.6, 2.9	**0.005**
FGF19 (ng/mL)						
POD 1	−387	−926, 152	0.154			
POD 3	−25	−325, 274	0.866			
POD 7	−50.3	−432, 532	0.834			
C4 (ng/mL)						
POD 1	−0.95	−3.9, 2.0	0.520			
POD 3	−0.498	−1.8, 0.78	0.433			
POD 7	−0.3	−1.6, 1.0	0.628			
Preoperative liver tests						
Bilirubin (µmol/L)	−0.4	−0.76, −0.02	0.**041**	−0.30	−0.62, 0.03	0.076
AST (IU/L)	0.08	−0.4, 0.6	0.767			
GGT (IU/L)	−0.00	−0.05, 0.03	0.665			
AP (IU/L)	−0.07	−0.19, 0.06	0.261			
Preoperative systemic inflammation						
CRP (mg/L)	−0.38	−0.73, −0.03	0.**036**			

*Note*: All continuous variables are per 1-unit increase. Follow-up time (time between the day of surgery and the last CT scan); postoperative day.

Significant P-values (<0.05) are in bold.

Abbreviations: AP, alkaline phosphatase; AST, aspartate aminotransferase; BMI, body mass index; C4, 7α-Hydroxy-4-cholesten-3-one; CRP, C-reactive protein; GGT, gamma-glutamyl transferase; pCCA, perihilar cholangiocarcinoma; POD, postoperative day; PVE, portal vein embolization.

### Dysregulated bile salt homeostasis is associated with poor clinical outcome

**FIGURE 5:**
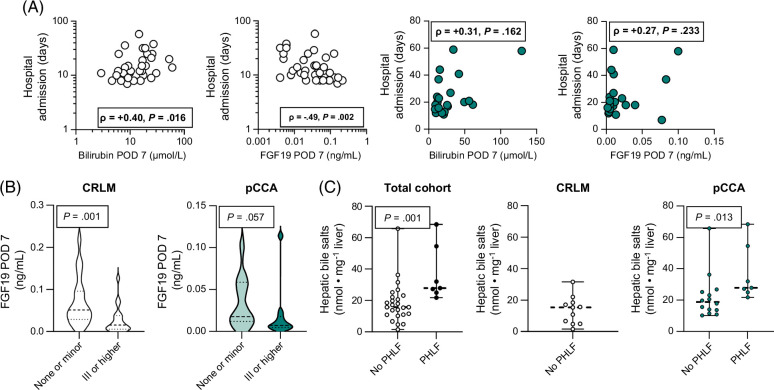
Dysregulated bile salt homeostasis is associated with postoperative complications. Bilirubin and FGF19 levels on POD 7 were studied in relation to length of hospital admission and postoperative complications. (A) Correlation between bilirubin or FGF19 levels on POD 7 versus length of hospital admission (days). (B) FGF19 levels on POD 7 in patients with minor or no postoperative complications versus patients with major postoperative complications (Clavien-Dindo classification grade III or higher) in patients with CRLM and pCCA. (C) Hepatic bile salts in the CRLM and pCCA groups who did not develop PHLF compared with those who developed PHLF. White and violin plots denote patients with CRLM and green-color denoted patients with pCCA, respectively. Correlations were tested using Spearman rank correlation. Differences between groups were evaluated using the Mann-Whitney *U* test. *p* values are depicted. Abbreviations: CRLM, colorectal liver metastases; pCCA, perihilar cholangiocarcinoma; PHLF, post-hepatectomy liver failure; POD, postoperative day.

FGF19 is considered anticholestatic and hepatoprotective in rodents, and recent clinical studies have shown promising beneficial effects in both patients with nonalcoholic steatohepatitis and primary sclerosing cholangitis treated with an engineered FGF19 analog.^31^,^32^,^33^,^34^ In CRLM controls, the length of hospital admission was positively related to bilirubin levels on POD 7 and negatively associated with FGF19 levels on POD 7 (Figure 5A). These relationships were not apparent in patients with pCCA. Furthermore, FGF19 levels on POD 7 were significantly lower in patients with CRLM with major postoperative complications and tended to be lower in patients with pCCA (Figure 5B). Administration of an engineered nontumorigenic variant of FGF19 effectively lowered liver bile salts in obstructive cholestasis (in rodents).^31^ Here, liver bile salts were significantly higher in patients with pCCA who developed PHLF, emphasizing perioperative treatment with FGF19 mimetics as a future therapeutic option (Figure 5C).

## DISCUSSION

In this study, we examined the bile salt-FGF19 axis in patients with pCCA preoperatively, studied the effect of partial liver resection with surgical restoration of bile flow on this axis, analyzed the impact on LR, and evaluated its relationship with postoperative complications. In general, our findings demonstrate preoperatively adapted gut-liver crosstalk in patients with pCCA, highlighted by near-to-full suppression of bile salt production through the classical synthetic route. In contrast to controls, partial liver resection in pCCA was not accompanied by postoperative elevation of circulating bile salts. Resection induced a steep decline in plasma bile salt and FGF19 levels within 24 hours and remained stable thereafter in pCCA, with the maintenance of an inverse correlation between FGF19 and C4, indicating sustained gut-liver crosstalk. We found that preoperative suppression of bile salt synthesis in pCCA normalized to levels similar to those in controls after 7 days, indicating fast recovery of hepatic metabolic function. Finally, postoperative bile salt levels 7 days after partial removal of the liver, corrected for age and preoperative bilirubin levels, were associated with LR.

Suppression of bile salt synthesis is an adaptive mechanism crucial to preventing intrahepatic bile salts from reaching toxic levels that trigger apoptosis and necrosis.^30^,^35^ In patients with intrahepatic or extrahepatic cholestatic (liver) diseases (eg, periampullary tumors, primary biliary cholangitis, alcoholic hepatitis, and biliary atresia), bile salt synthesis is suppressed, likely as a cytoprotective mechanism by the inhibitory actions of FGF19.^15^,^16^,^17^,^18^,^36^ Consistent with these reports, bile salt homeostasis before resection was markedly altered in pCCA in the current study, as highlighted by elevated systemic bile salt and FGF19 levels. Under physiological conditions, FGF19 is produced in an FXR-stimulated fashion in the terminal ileum; however, cholestatic conditions (eg, primary biliary cholangitis and alcoholic hepatitis) trigger hepatic FGF19 production, which likely suppresses bile salt synthesis through auto- or paracrine signaling.^15^,^37^

The striking finding of this study was the lack of postoperative bile salt elevation in the pCCA group, in contrast to the gradually rising bile salt levels up to day 7 in the controls. The latter confirmed findings in a previously reported cohort of patients with CRLM and preclinical models.^19^,^29^,^38^,^39^ Because postoperative plasma bile salt elevations could reflect the initiation of LR in controls, the absence of an increase in postoperative bile salt levels could be a matter of concern in pCCA. The absence of postoperative bile salt elevation in patients with pCCA could be explained by a decreased pool of cycling bile salts. In line with this, postoperative FGF19 levels were even lower in patients with pCCA compared with CRLM controls and healthy volunteers,^27^ indicating reduced bile salt uptake across the ileal border. However, it cannot be ruled out that changes in the postoperative biliary anatomy of pCCA underlie the lack of postoperative bile salt elevation. Specifically, hepaticojejunostomy channels hepatic bile directly into the small intestine, without the requirement for sphincter of Oddi relaxation. Hence, a continuous jejunal influx of hepatic bile occurs, and enterohepatic recirculation of bile salts is more frequent. If the extraction capacity of the liver remnant is sufficient in the pCCA group, no systemic spill-over or postoperative elevation would occur. The ultimate liver volume recovery was comparable between patients with pCCA and controls. Patients with pCCA were older at the time of surgery and had higher preoperative bilirubin levels. Multivariable regression analysis of the total cohort revealed that postoperative bile salt levels on day 7 after surgery, corrected for age at the time of surgery and preoperative bilirubin levels, were significantly associated with LR. In the context of LR in pCCA, the complexity of the disease (eg, preoperative cholestasis and/or preoperative biliary drainage, altered dynamics of enterohepatic circulation after surgery, and inflammatory status) is likely to affect the hepatic regenerative response.

The postoperative rise of bile salts in CRLM, considered a normal physiological phenomenon, could imply metabolic overload in the remnant liver and in effect trigger LR, a theory (“hepatostat”) coined earlier by Fausto et al.^40^ Mechanistically, the postoperative increase in systemic bile salts could be attributed to maintained bile flow and portal bile salt supply, but a decreased extraction capacity due to a lower RLV. Indeed, only in patients with CRLM, the percentage of RLV was negatively associated with postoperative bile salt levels.^41^,^42^,^43^,^44^,^45^,^46^,^47^ Moreover, postoperative repression of the Na+-taurocholate cotransporting polypeptide (*NTCP*), responsible for the basolateral uptake of bile salts, could contribute to the maintenance of elevated systemic bile salt levels in the postoperative phase.^46^ In addition, the postoperative rise of systemic bile salts in CRLM controls was not related to C4, suggesting that de novo bile salt production did not contribute to the elevated bile salt levels in the postoperative phase.

Absolute preoperative values of bile salts, FGF19, and C4 in the CRLM control group, were similar as previously reported by us for healthy controls.^27^ As expected, hepatic bile salts in the pCCA group were higher than those in the control group. Because de novo bile salt production was not related to systemic and hepatic bile salts in pCCA, unlike in controls, it is likely that elevated hepatic bile salts could be explained by reduced *BSEP* expression or reflect bile accumulation in the volumetrically expanded biliary tree.^48^,^49^ Interestingly, hepatic bile salts strongly predicted hyperbilirubinemia 7 days after resection, and levels were even higher in patients who developed PHLF. This strengthens our previous finding that hepatic bile salt accumulation precedes the development of hyperbilirubinemia and suggests that it might predict metabolic overload.^19^ Preoperative strategies to normalize hepatic bile salts next to biliary drainage could be promising in the future.

Remarkably, postoperative C4 in pCCA was restored to levels similar to those in CRLM controls on day 7, an observation not previously reported in patients. The dynamics of C4 before and after partial liver resection in controls were consistent with similar findings in preclinical studies.^9^,^29^,^50^ C4 decreases strongly after postoperative loss of liver volume and then rebounds to preoperative levels. The exact mechanism for the initial drop in the classical route of bile salt synthesis, apart from the removal of a large part of the source of C4 after partial liver resection, has not been entirely clarified.^51^,^52^ Zhang et al^50^ demonstrated that the c-Jun N-terminal Kinase signaling pathway might contribute to transcriptional downregulation of *Cyp7a1* after partial hepatectomized mice. This signal transduction pathway can be activated by FGF receptor 4 (FGFR4) stimulation, the cognate receptor of Fgf15/FGF19.^53^,^54^ Therefore, postoperative FGF19 signaling could play a role in the repression of bile salt synthesis after partial liver resection. It is imperative to control bile salt synthesis during LR, as *Cyp7a1* overexpression is associated with increased hepatic bile salts and impaired LR, increased apoptosis, and liver injury.^10^,^50^ Since postoperative C4 levels were negatively correlated with international normalized ratio in the current study and low levels were observed in patients with PHLF, this suggests that restored bile salt synthesis might reflect the hepatic metabolic reserve capacity during liver regrowth. In line with this statement, we showed that remnant liver negatively affected postoperative C4, which is related to the fact that a smaller remnant liver is associated with postoperative liver failure.^26^

The strength of this study was the pretest and posttest design with partial liver resection as an intervention. In addition, multiple blood samplings to study alterations in the bile salt-FGF19 axis and bile salt synthesis in the long postoperative period strengthened this study. Despite the better translatability of this study in contrast to preclinical studies, a few limitations of this study need to be addressed. First, CT volumetrics were not performed at predefined time points. However, the follow-up time did not influence the regression analyses and was comparable between patient cohorts. In addition, regeneration takes place mainly in the first postoperative week, as shown in patients who underwent major partial liver resection for either living liver donation or tumor removal without the preoperative existence of chronic liver disease or biliary obstruction.^55^ Furthermore, although striking, the findings that (i) C4 levels are significantly lower in patients with PHLF and (ii) FGF19 levels are lower in patients with postoperative major complications need to be taken with caution because this study was not designed to evaluate clinical outcomes. In this regard, a small sample size in the multivariable analysis also needs to be kept in mind.

The findings from this study provide insights into perioperative bile salt homeostasis in patients with pCCA after partial hepatectomy. This is of particular importance because surgical intervention in pCCA is associated with significant postoperative mortality, primarily related to PHLF. Moreover, monitoring bile salts and C4 in clinical practice is feasible, and pharmacological options such as various FXR modulators (eg, Ocaliva) and FGF19 mimetics are currently used clinically or evaluated in clinical trials, respectively. Thus, modulation of bile salt homeostasis may have potential in the clinical management of patients with pCCA.

In conclusion, this study yielded several important findings: (i) gut-liver crosstalk by the bile salt-FGF19 axis is altered preoperatively and likely protective in pCCA; (ii) partial liver resection impacts on this axis such that postoperative bile salts and FGF19 are low, and only bile salt levels on POD 7 are an independent predictor of LR; (iii) bile salt synthesis through the classical route appears to normalize after 7 days in pCCA, suggesting restoration of hepatic metabolic capacity within the first week; (iv) liver bile salts predict hyperbilirubinemia in the postoperative phase and elevated hepatic bile salt content might be involved in the development of PHLF; (v) patients with PHLF have low C4 levels; and (vi) low postoperative FGF19 levels could be an indicator of postoperative liver dysfunction and could lead to consideration of FGF19 supplementation. Further research on the control of bile salts and bile salt synthesis in the postoperative phase could clarify human LR in pCCA and contribute to potential therapeutic options. Careful monitoring of circulating bile salts and C4 levels may be useful in guiding clinical management. The technical requirements to analyze C4 in particular, is time-consuming and require dedicated analytical equipment and personnel expertise. At present, this has no added value over the currently available clinical markers for postoperative recovery of liver functions. However, the current data justify further investigation of the relevance of bile salts and C4 plasma values in the assessment of postoperative liver function. FGF19 mimetics should be considered for protection against hepatocellular injury in pCCA.

## Supplementary Material

SUPPLEMENTARY MATERIAL
