# Supplementary material for: Partial liver resection alters the bile salt-FGF19 axis in patients with perihilar cholangiocarcinoma: Implications for liver regeneration
Source: Hepatol Commun. 2024 Jun 5;8(6):e0445. doi: 10.1097/HC9.0000000000000445 (PMC11155560; doi:10.1097/HC9.0000000000000445)

**SUPPLEMENTARY FILES**

| Table S1. CT-liver volumetry | | | |
| --- | --- | --- | --- |
| (calculated) CT-volumetric item | **CRLM (n = 35)**  **mean (± SD)** | **pCCA (n = 17)**  **mean (± SD)** | ***P*-value** |
| Preoperative TLV (mL) | **1571 [1407 to 1878]** | 1821 [1515 to 2405] | **.077** |
| Anticipated RLV (mL) | 567 [ 492 to 923] | 931 [773 to 1494] | **.008** |
| Anticipated RLV (%) | 38 [29 to 65] | 55 [35 to 67] | .203 |
| Postoperative FLV (mL) | 1200 [1020 to 1482] | 1503 [1090 to 1781] | **.042** |
| Postoperative FLV^±^ (%) | 75 [65 to 89] | 79 [60 to 90] | .776 |
| LR-index*^±^ (%) | 82 [32 to 127] | 42 [23 to 87] | .072 |
| ^±^ postoperative FLV: [(FLV/TLV-TuV)]*100 and LR-index: (pFLRV – RLV)*100; *without abscesses; TLV, total liver volume; RLV, remnant liver volume; FLV, functional liver volume; LR, Liver regeneration | | | |

| Table S2. Type of partial liver resection | |
| --- | --- |
| CRLM (n = 43) | **pCCA (n = 23)** |
|  | Trisectionectomy right (n = 6) |
| Extended hemihepatectomy left (n = 2) | Trisectionectomy left (n = 1) |
| Extended hemihepatectomy right (n = 15) | Extended hemihepatectomy left (n = 9) |
| Hemihepatectomy left (n = 4) | Extended hemihepatectomy right (n = 3) |
| Hemihepatectomy right (n = 16) | Hemihepatectomy left (n = 1) |
| Multiple segments resection (n = 6) | Hemihepatectomy right (n = 2) |
|  | Multiple segment resection (n = 1) |

| **Table S3. Differences of FGF19 and bile salts over time between CRLM and pCCA patients.** | | | | | | |
| --- | --- | --- | --- | --- | --- | --- |
| **Variable** | **FGF19 (ng/mL)** | | | **Bile salts (µmol/L)** | | |
|  | Estimate | 95% CI | *P*-value | Estimate | 95% CI | *P*-value |
| *Intercept* | .239 | .195, .285 | **<.001** | 25.5 | 18.9, 32.1 | **<.001** |
| *Group* (CRLM) | -.163 | -.220, -.107 | **<.001** | -16.8 | -24.3, -8.6 | **<.001** |
| *POD 1 vs BL* | -.192 | -.261, -.123 | **<.001** | -21.7 | -31.6, -11.8 | **<.001** |
| *POD 3 vs BL* | -.218 | -.283, -.153 | **<.001** | -19.5 | -28.9, -10.1 | **<.001** |
| *POD 7 vs BL* | -.212 | -.277, -.147 | **<.001** | -19.2 | -31.7, -11.8 | **<.001** |
| *Group * POD 1 vs BL* | ,152 | .067, .236 | **<.001** | 20.6 | 8.0, 32.5 | **.001** |
| *Group * POD 3 vs BL* | ,191 | ,110, .273 | **<.001** | 27.8 | 16.0, 39.9 | **<.001** |
| *Group * POD 7 vs BL* | ,186 | .105, .267 | **<.001** | 28.2 | 16.4, 39.9 | **<.001** |
| Baseline (BL, preoperative) as reference time point; Group (pCCA as reference group); Explanation of the model: Intercept – mean value in pCCA patients at baseline; Group (CRLM) – difference from intercept if in CRLM patients; POD 1 / 3/ 7 *vs* BL – difference from baseline in pCCA patients at POD 1, 3 and 7; Group * POD 1/3/ or 7 *vs* BL – interaction effect, representing the difference between pCCA and CRLM patients, change from baseline at POD 1, 3 and 7. | | | | | | |

| Table S4. Patient characteristics | | | |
| --- | --- | --- | --- |
| Characteristic | **Without CT-volumetry (n = 20)** | **With CT-volumetry**  **(n = 46)** | ***P*-value** |
| Sex |  |  |  |
| Male | 17 | 25 | **0.025** |
| Female | 3 | 21 |  |
| Mean age (years) | 62 ± 12 | 60 ± 11 | 0.627 |
| Median BMI (kg/m^2^) | 24.4 [21.5 to 28.3] | 25.9 [22.3 to 30.4] | 0.357 |
| *ASA 3 or 4* |  |  |  |
| No | 8 | 20 | 1.000 |
| Yes | 12 | 26 |  |
| *Preoperative laboratory values* |  |  |  |
| Bilirubin (µmol/L) | 8 [6 to 15] | 11 [5 to 19] | 0.399 |
| AST (IU/L) | 39 [31 to 68] | 38 [30 to 50] | 0.764 |
| γGT (IU/L) | 105 [40 to 466]  (n = 19) | 143 [62 to 405]  (n = 44) | 0.747 |
| AP (IU/L) | 142 [78 to 332]  (n = 19) | 146 [98 to 293]  (n = 44) | 0.579 |
| Albumin (g/L) | 4.2 [3.7 to 4.5] (n = 14) | 3.9 [3.3 to 4.3] (n = 32) | 0.090 |
| CRP (mg/L) | 6 [2 to 73] | 12 [4 to 21] (n = 44) | 0.533 |
| INR | 0.97 [0.90 to 1.13] | 1.00 [0.93 to 1.08]  (n = 45) | 0.363 |
| *Preoperative biliary drainage* |  |  |  |
| No | 17 | 31 | 0.229 |
| Yes | 3 | 15 |  |
| *PVE* |  |  |  |
| No | 15 | 33 | 1.000 |
| Yes | 5 | 12 |  |
| *Postoperative clinical outcome* |  |  |  |
| Posthepatectomy liver failure (PHLF) |  |  |  |
| No | 19 | 40 | 0.666 |
| Yes | 1 | 6 |  |
| 90-day mortality |  |  |  |
| No | 19 | 42 | 1.000 |
| Yes | 1 | 3 |  |
| Hospital admission (days) | 12 [8 to 20] | 16 [11 to 26] | 0.092 |
| Major surgical morbidity (C-D III-V) |  |  |  |
| No | 14 | 23 | 0.180 |
| Yes | 6 | 23 |  |
| Values depicted as mean SD or median [IQR]. Student’s *t*-test, Chi square or Mann-Whitney U test were used to test differences between groups. CRLM, colorectal liver metastases; pCCA, perihilar cholangiocarcinoma; BMI, body mass index; ASA, American Society of Anesthesiologists; AST, aspartate aminotransferase; GGT, gamma glutamyl transferase; AP, alkaline phosphatase; CRP, C-reactive protein; INR, International normalized ratio; PVE, portal vein embolization; C-D, Clavien-Dindo | | | |


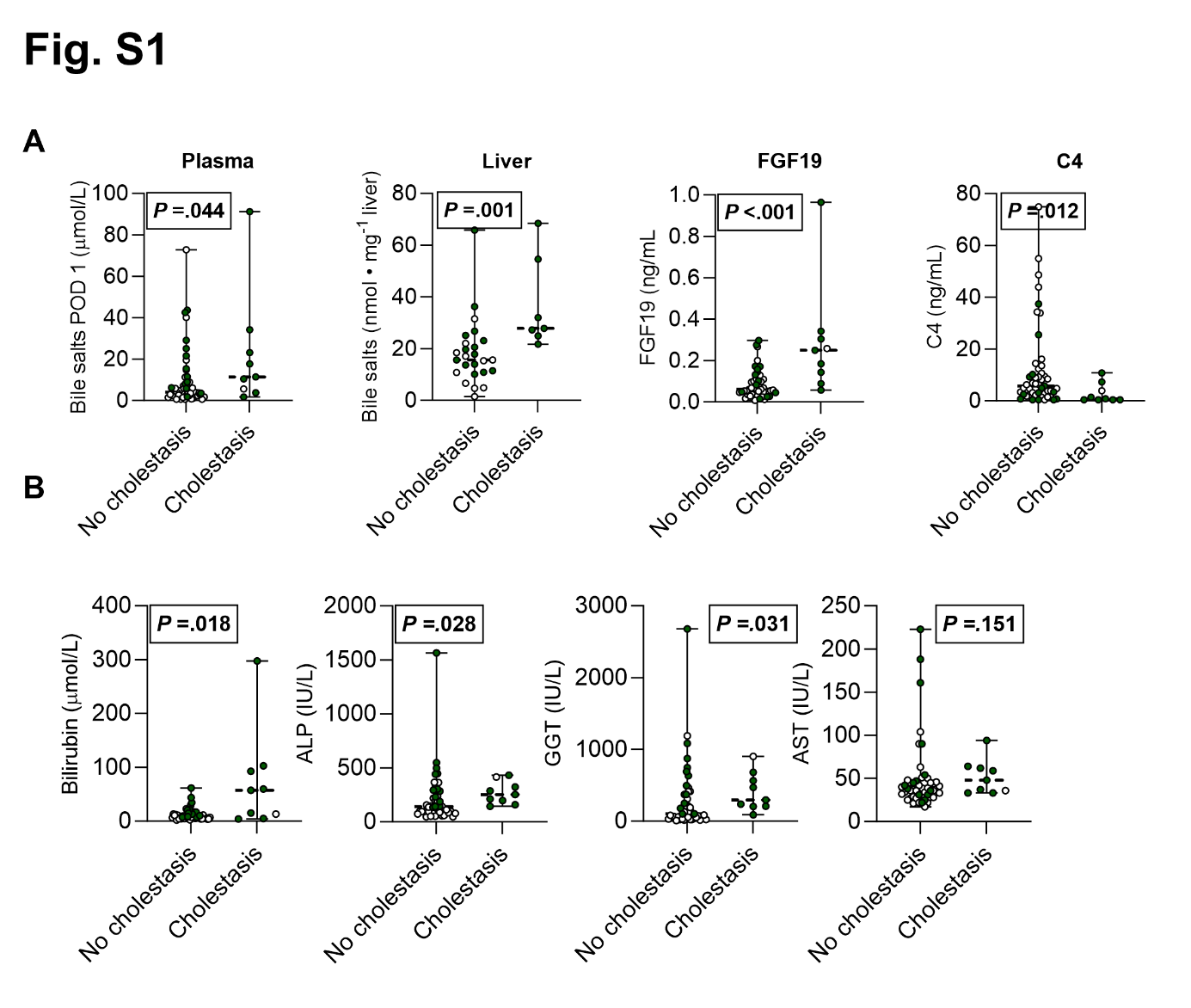


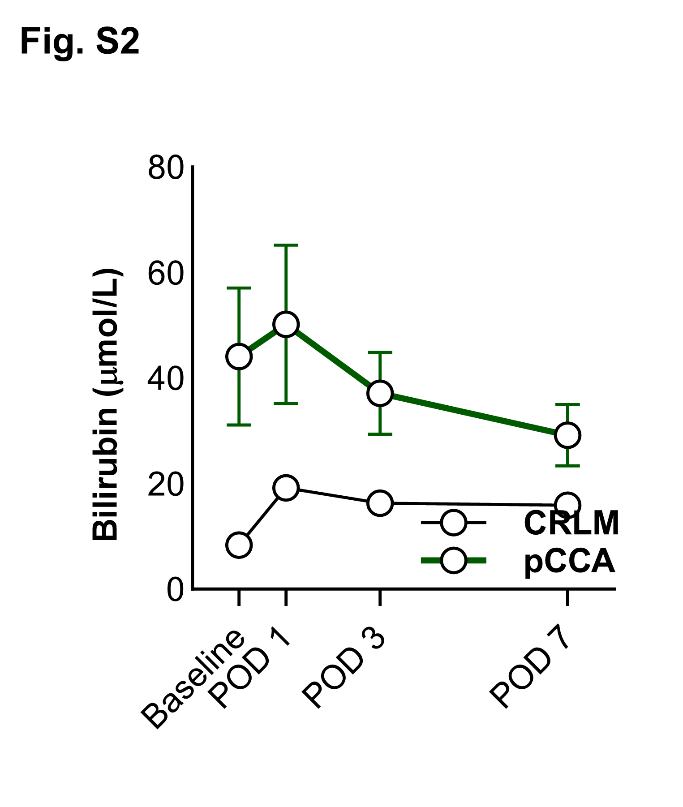

Supplement: SUPPLEMENTARY MATERIAL [file hc9-8-e0445-s001.docx]
